# Urban Refugee Youth’s Recommendations for Sexual and Mental Health Promotion: Qualitative Insights From Kampala, Uganda

**DOI:** 10.1177/15248399251327951

**Published:** 2025-03-25

**Authors:** Isabel G. Sternthal, Frannie MacKenzie, Carmen H. Logie, Moses Okumu, Suliat Fehintola Akinwande, Bay Bahri, Robert Hakiza, Daniel Kibuuka Musoke, Brenda Katisi, Aidah Nakitende, Peter Kyambadde

**Affiliations:** 1 University of Toronto, Toronto, Ontario, Canada; 2 Women’s College Hospital, Toronto, Ontario, Canada; 3 United Nations University Institute for Water, Environment & Health, Hamilton, Ontario, Canada; 4 The University of North Carolina, Chapel Hill, NC, USA; 5 Young African Refugees for Integral Development (YARID), Kampala, Uganda; 6 International Research Consortium, Kampala, Uganda; 7 Ministry of Health, Kampala, Uganda; 8 Most At Risk Populations Initiative, Mulago Hospital, Kampala, Uganda

**Keywords:** refugee health, urban refugees, refugee youth, health promotion, youth empowerment, Uganda, Kampala, sexual reproductive health, mental health

## Abstract

Refugee youth in Kampala, Uganda, face a unique sexual reproductive health and mental health risk environment requiring focused interventions. Resource limitations and access barriers complicate the provision of relevant supports. Few studies have engaged refugee youth’s recommendations for satisfying their sexual and mental health needs. This cross-sectional, qualitative study aimed to identify urban refugee youth’s sexual and mental health promotion preferences. We administered a structured survey to refugee youth in Kampala (n = 54) between July and November 2023 using the qualitative data collection platform, Sensemaker and analyzed survey responses using inductive thematic analysis (ITA). Two themes emerged: health promotion and youth empowerment. Participants proposed sexual and mental health promotion recommendations directed at distinct stakeholder groups including policymakers/service providers and peers. There were several points of overlap between the sexual and mental health promotion recommendations, including calls for counseling services, health education, and employment opportunities. Youth empowerment was a central theme underwriting both sexual and mental health recommendations. By differentiating between recommendations directed at distinct stakeholder groups, this study identified opportunities for non-governmental actors to contribute to promoting the sexual and mental health of refugee youth in Kampala. Participant insights show how engagement with urban refugee youth’s health promotion recommendations can empower youth and ensure that service design and delivery is consistent with their knowledge, needs, and preferences.

In 2022, the United Nations (UN) estimated the size of the global refugee population at 108.4 million people, with 76% of refugees hosted in low and middle-income countries (LMICs; UN High Commissioner for Refugees [UNHCR], 2022). Despite growing urbanization, the health promotion needs of urban refugees in LMICs are understudied. A 2024 scoping review identified a gap in research on the sexual reproductive health (SRH) needs of urban refugee youth in LMICs, and more broadly, urban refugees in African LMICs (Logie et al., 2024). In Uganda, a country with a refugee population of 1.5 million (UNHCR, 2022), an “open door refugee policy” entitles refugees who do not require humanitarian assistance to live in urban centers (Ahimbisibwe, 2019). Kampala, Uganda’s largest urban center, is home to more than 95,000 refugees (UNHCR, 2023) who face extensive resource limitations and administrative barriers to health care (Walnycki et al., 2019). Due in part to reduced humanitarian assistance (Walnycki et al., 2019), refugee youth, a demographic who make up 26% of Kampala’s refugee population (UNHCR, 2023), are at an elevated risk of negative SRH outcomes, including intimate partner violence (IPV; Ogbonnaya et al., 2022) and HIV vulnerabilities (Logie et al., 2022), and negative mental health outcomes, including depression, posttraumatic stress disorder (PTSD) (Hettich et al., 2020), and substance use as a coping mechanism (Bahati et al., 2023). Although these challenges present a need for tailored interventions, knowledge gaps persist regarding the sexual and mental health promotion preferences of refugee youth in Kampala (Logie et al., 2022). Through this cross-sectional, qualitative research brief, we aim to inform researchers, policy makers, and practitioners of urban refugee youth’s health promotion directives.

## Method

### Data Collection

We collected data using Sensemaker, a web-based data collection tool developed in collaboration with a Sensemaker consultant. The platform invited participants to share a narrative focused on SRH and mental health promotion through structured questions such as: “What are your recommendations for improving young people’s sexual health?” and “What are your recommendations for improving young people’s mental health?” A team of research assistants (*n* = 8), trained in qualitative methods, administered the tool in the participant’s language of choice (English, French, Swahili, Kirundi, and Kinyarwanda) in person, using a tablet. Research assistants audio-recorded participant responses on the Sensemaker platform, transcribed, and translated responses verbatim. We purposively sampled participants (*n* = 54) from an existing cohort (Logie et al., 2023). Cohort eligibility included refugee youth aged 16 to 24 living in one of five informal settlements in Kampala (Rubaga, Kansanga, Kabalagala, Nsambya, and Katwe). A team of peer navigators (*n* = 12) recruited eligible participants over the phone. The University of Toronto (#37496), the Mildmay Uganda Research Ethics Committee (#MUREC-2021-41) and the Uganda National Council for Science & Technology (SS1021ES) granted research ethics board approval. Data collection occurred between July and November 2023.

### Data Analysis

We analyzed the Sensemaker transcripts using inductive thematic analysis (ITA; Braun & Clarke, 2006). After creating a preliminary code book based on an open reading of the data, we consolidated related codes into themes in an iterative process of data (re)organization and dialogue among the research team members. I.G.S. thematically coded data in consultation with F.M., C.H.L., and S.F.A., qualitative methodologists with extensive global health research experience.

## Results

The research team collected descriptive statistics for all participants (Table 1).

**Table 1:** Sociodemographic Information of Urban Refugee Youth Participants in Kampala, Uganda (*n* = 54), 2023

*Demographic*	*n (%)*
Gender	
Woman	26 (48)
Man	28 (52)
Age (years)	
17	2 (4)
18	8 (15)
19	7 (13)
20	4 (7)
21	5 (9)
22	5 (9)
23	7 (13)
24	16 (30)
*M* age	21.31
Age range	7
*SD*	2.41
Place of birth	
Burundi	8 (15)
DRC	41 (76)
South Sudan	1 (2)
Tanzania	1 (2)
Uganda	3 (6)
Education level	
Less than secondary school	30 (56)
Completed secondary	17 (31)
Attended some tertiary school	1 (2)
Completed tertiary institution	4 (7)
Completed university (bachelor’s)	2 (4)
Employment status	
Student	12 (22)
Unemployed	24 (44)
Self-employed	11 (20)
Paid employment	7 (13)
Relationship status	
No current partner	26 (48)
Casual dating	10 (19)
Dating, not living together	11 (20)
Living together	1 (2)
Married	6 (11)
Number of children	
0	44 (81)
1	6 (11)
2	3 (6)
3	0 (0)
4	1 (2)

Two overarching themes emerged: health promotion and youth empowerment. Representative quotations are provided below to illustrate each theme.

### Health Promotion

The theme of health promotion contains SRH promotion recommendations and mental health promotion recommendations. We distinguish between health promotion recommendations directed toward policy makers and service providers, and toward peers.

#### SRH Promotion Recommendations

##### SRH promotion recommendations for policy makers and service providers

Participants called on policy makers and service providers to address structural determinants of SRH, such as unemployment.

[Youth] sexual health is related to their lack of work because people sexually exploit them. If we can get them skills, maybe their sexual health can be improved because when someone is busy and they are working themselves it’s not easy for someone else to come in [and exploit them]. (Man, 24)

SRH service provider recommendations were varied. Some participants requested free SRH materials, including condoms, HIV tests, and menstrual products.

First of all, for us girls, I think they should make period products free. Because some of us don’t get access to them. The pads . . . some of them [are] expensive. Other girls like me don’t get access to them because [we] got no money. (Woman, 17)

Others expressed support for one-on-one and group SRH counseling services. Participants suggested that both counseling services and SRH educational events could occur in a designated sexual health center, enabling opportunities for both single-gender and co-educational events.

A community space for example . . . having a room that is just for this particular issue [SRH] could be good. [There should be] one for boys, one for girls, [and] one shared space for youth where they can talk about this [SRH]. (Man, 24)

Proposed formats for SRH education included seminars led by knowledgeable speakers on topics such as condom use, HIV/sexually transmitted infections (STI) risk mitigation, and service options.

##### SRH promotion recommendations for peers

When sharing SRH recommendations for their peers, some participants encouraged youth to “test for HIV [and] use condoms during unplanned sexual intercourse” (Woman, 23), whereas others encouraged their peers to practice abstinence.

I would like to advise young people like me to stop having sex because before [the right] age it [will] destroy their life. (Man, 18)

#### Mental Health Promotion Recommendations

##### Mental health promotion recommendations for policy makers and service providers

Participants called on policy makers and service providers to increase access to free, conveniently located/online, and trauma-informed counseling services.

I think there should be free counseling for everybody . . . somebody to encourage them into life again, tell them not to give up yet. (Woman, 17)

To address a shortage of qualified counselors, some participants suggested training youth as peer counselors.

Professional counselors have to train the peer counselors so that they can also help the community and make sure young people are accessing counseling easily. (Man, 24)

Youth also expressed interest in working with substance use counselors equipped to discuss drug and alcohol use. Participants noted that maladaptive substance use might be linked to idleness, which could be addressed by providing occupational training.

Nowadays young people are abusively using drugs [and] alcohol. Young people need jobs. A person like me, if you give me a job, I will work. (Woman, 21)

Others suggested that sharing information about addiction could help to prevent unhealthy substance use.

##### Mental health promotion recommendations for peers

Participants encouraged peers to stay busy as a coping strategy, either by engaging in recreational activities like football or by seeking employment.

Some young people have started using drugs . . . and it’s not good at all. They should look for some occupation so that mentally they stop soaking [up] stress. (Man, 18)

Furthermore, participants encouraged their peers to challenge mental health stigma and noted its connection to SRH stigma.

You can advise [other youth] to spend more time with people [and] to get [them] to have conversations about their health problems and sexual problems so that they don’t keep it to themselves. That could help them mentally. (Man, 18)

### Youth Empowerment

Participants emphasized the importance of empowering youth to take an active role in promoting their SRH and mental health. Some raised employment as a central mode of youth empowerment, with potential to bolster participants’ well-being.

Youths should be empowered by providing employment opportunities to them which will keep them hopeful for a better tomorrow and give them purpose to live because we all live like [we] have no assurance of tomorrow. (Man, 23)

Reflections on the importance of affirming youth’s bodily autonomy in health promotion were prominent. One participant reflected on how their sense of bodily autonomy was tied to their ability to express their gender and sexual identity.

I’m talking about owning choices and making decisions about my own body. When it comes to dressing up, what I really want to identify as, when it comes to what my sexual orientation and gender identity would want to be. . .[I] want to own that . . . It should be entirely me, not my parent or any guardian, or anyone. (Man, 24)

Finally, participant reflections suggested that the experience of participating in this study was a source of empowerment with positive mental health impacts.

I would like to tell you thank you for this program [interview], because actually, getting this all out of me has not been easy but at least now I feel like something heavy has gone out of me. So thank you. (Woman, 17)

Participants suggested that the sharing of lived experiences and preferences through an academic study impacted their mental health. Some described feeling empowered by the knowledge that, “someone somewhere knows what I am going through and also I add a voice to the many youths [who] need assistance . . . especially those of us in the slums.” (Man, 23)

## Discussion

We identified urban refugee youth’s recommendations for promoting their SRH and mental health. SRH recommendations for policy makers and service providers concerned addressing determinants of SRH and providing SRH services and information, whereas recommendations directed toward peers concerned SRH behaviors and attitudes. Mental health promotion recommendations for policy makers and service providers included calls to address mental health determinants and outcomes through services such as counseling. Recommendations directed toward peers concerned mental health attitudes and coping strategies. Participants suggested that securing employment, affirming their bodily autonomy, and sharing their experiences could be sources of youth empowerment. A novel aspect of our study is the finding that some urban refugee youth perceived community-based research as a health promotion practice which helped to affirm the value of their perspectives. Further research should consider this dynamic, previously documented by Lajoie et al. (2020) in the context of Canadian, community-based mental health research, among urban refugee youth in LMICs.

Our findings suggest that there is substantial overlap between the SRH and mental health promotion recommendations in existing literature and the preferences of refugee youth in Kampala. Requests for SRH materials, counseling services, and health education are consistent with published recommendations (Logie et al., 2022), as are calls to address urban refugee youth unemployment as a driver of poor SRH and mental health (Shand et al., 2021). Participants’ endorsement of abstinence is a notable point of departure from the established finding that abstinence-only education is ineffective as a harm reduction method (de Haas et al., 2017).

The authors wish to note that results may not be generalizable to other urban refugee youth populations. Purposive participant recruitment may have impacted the representativeness of the sample.

## Implications for Practice

The results of this study can inform several aspects of health promotion practice with respect to refugee youth in Kampala. First, multiple recommendations were relevant to both SRH and mental health promotion, including calls to increase access to counseling services, health education, and employment opportunities. These points of overlap suggest the potential to further integrate SRH and mental health promotion interventions.

Furthermore, by distinguishing between recommendations for policy makers and service providers and recommendations for peers, our findings reveal opportunities for diverse stakeholders to contribute to promoting the SRH and mental health of refugee youth in Kampala. In the absence of robust, governmental humanitarian aid (Walnycki et al., 2019), these recommendations can help to guide the distribution of health promotion initiatives among appropriate non-governmental actors. For instance, consistent with prior support for peer-led SRH and mental health services (UNHCR, 2016), the participants’ recommendation of introducing peer counseling services can help to promote youth mental health. Collaboration between non-governmental actors and governmental institutions could enhance intervention reach and sustainability.

Finally, this study affirms the importance of engaging refugee youth knowledge and preferences in health promotion efforts, as previously reported (UNHCR, 2016). Participants’ reflections on contributing to the study suggest that refugee youth are keen to engage with service design and delivery efforts. Direct engagement with urban refugee youth’s health promotion recommendations can help to ensure that intervention design reflects their needs and preferences.
